# Supplementary material for: Cell-type-specific mRNA transcription and degradation kinetics in zebrafish embryogenesis from metabolically labeled single-cell RNA-seq
Source: Nat Commun. 2024 Apr 10;15:3104. doi: 10.1038/s41467-024-47290-9 (PMC11006943; doi:10.1038/s41467-024-47290-9)
Supplement: Supplementary file 1 — Supplementary Information [file 41467_2024_47290_MOESM1_ESM.pdf]

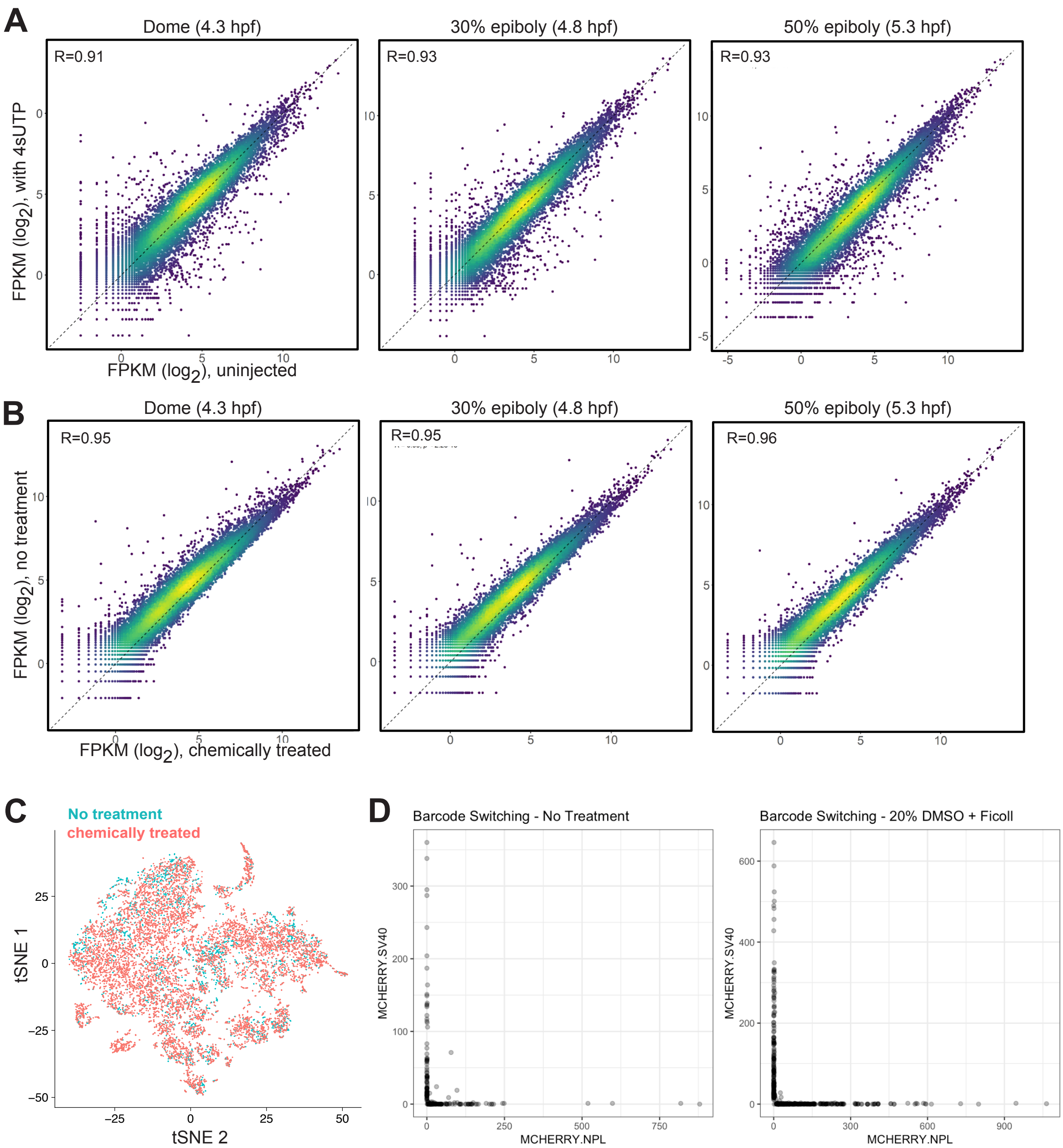

A
Dome (4.3 hpf)
30% epiboly (4.8 hpf)
50% epiboly (5.3 hpf)
R=0.91
R=0.93
R=0.93
FPKM (log2), with 4sUTP
FPKM (log2), uninjected
B
Dome (4.3 hpf)
30% epiboly (4.8 hpf)
50% epiboly (5.3 hpf)
R=0.95
R=0.95
R=0.96
FPKM (log2), no treatment
FPKM (log2), chemically treated
C
No treatment
chemically treated
tSNE 1
tSNE 2
D
Barcode Switching - No Treatment
Barcode Switching - 20% DMSO + Ficoll
MCHERRY.SV40
MCHERRY.NPL

Supplementary Figure 1: combining scRNA-Seq, mRNA metabolic labeling and nucleotide conversion in zebrafish embryos

**(A-B)** Scatter plots of RNA expression ($log_2$) in 3 developmental stages (left to right: dome, 4.3 hpf; 30% epiboly, 4.8 hpf; 50% epiboly, 5.3 hpf). Colors represent density (yellow=high density; blue=low density). Pearson R values are indicated. Expression was calculated as the normalized sum of all UMIs in cells within each stage. **(A)** scRNA-Seq results of uninjected embryos (x-axis; data from [1]) or embryos that were injected with 4sUTP at the 1-cell stage (y-axis; this study). **(B)** scRNA-Seq results of embryos that were injected with 4sUTP at the 1-cell stage, and were either chemically treated (x-axis) or not treated (y-axis). **(C)** tSNE projection of single cells from embryos that were injected with 4sUTP at the 1-cell stage, and were either chemically treated (red) or not treated (cyan). **(D)** Scatter plots of UMI counts associated with each single-cell in 3 out of 5 of our samples containing a mixture of embryos that were randomly injected with one of two mRNA species (bearing a different C-terminal sequence), labeled as MCHERRY.NPL (x-axis) and MCHERRY.SV40 (y-axis). Samples were either chemically treated (right) or not treated (left). Less than 0.05% of cells in both converted and non-converted samples were associated with reads from both mCherry species, indicating that conversion did not interfere with RNA attachment to beads and did not increase barcode mixing.

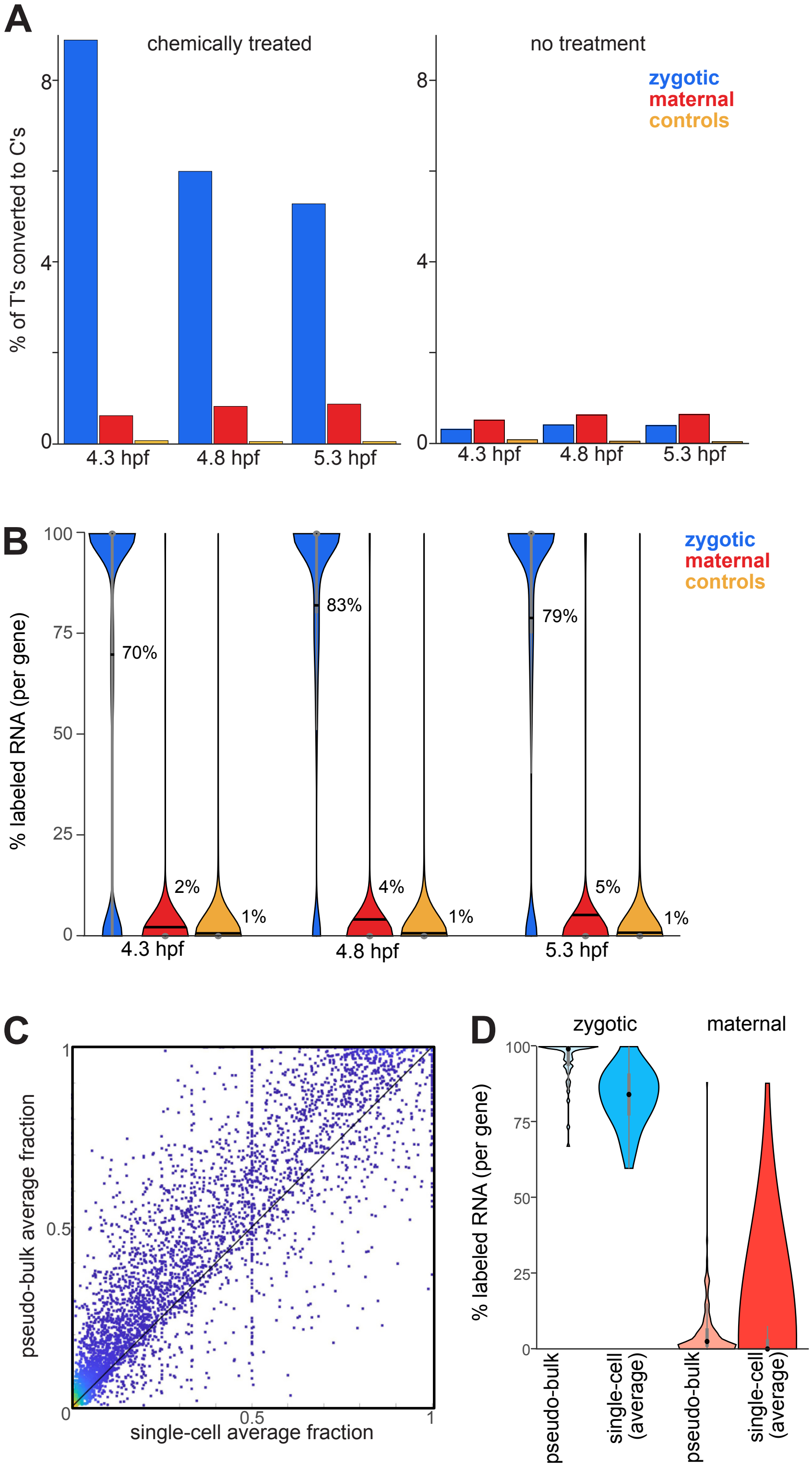

A
chemically treated
no treatment
% of T's converted to C's
zygotic
maternal
controls
4.3 hpf
4.8 hpf
5.3 hpf
B
% labeled RNA (per gene)
70%
2%
1%
83%
4%
1%
79%
5%
1%
4.3 hpf
4.8 hpf
5.3 hpf
zygotic
maternal
controls
C
pseudo-bulk average fraction
single-cell average fraction
D
zygotic
maternal
% labeled RNA (per gene)
pseudo-bulk
single-cell (average)
pseudo-bulk
single-cell (average)

Supplementary Figure 2: distinguishing maternal and zygotic mRNAs in scRNA-Seq metabolic labeling

**(A)** Fraction of T bases sequenced as C (y-axis), within 3 temporal samples (x-axis), when applying chemical treatment (left) or without treatment (right). Fractions calculated for subsets of 110 zygotic (blue), 332 maternal (red), and 3 injected control mRNAs (yellow). As label was introduced in the 1-cell stage, transcripts from zygotic genes (blue) are expected to be fully labeled in all samples. Differences in converted fraction can arise from different incorporation efficiencies between samples, position-specific incorporation rates, genetic polymorphism and other confounding effects. **(B)** GRAND-SLAM estimates of per-gene percent of labeled RNA (y-axis) within single cells. Central dot is median; box bounds are 25th and 75th percentiles, upper and lower limits of whiskers are 1.5x interquartile ranges. Values outside of the upper and lower limits are defined as outliers, n=100 zygotic (blue), 288 maternal (red) and 3 injected control mRNAs (yellow). Some of the genes analyzed in panel (E) were not analyzed by GRAND-SLAM due to relatively low expression within single cells. **(C)** Fraction of zygotic mRNA assigned to genes by either average of single cell estimations (x-axis) or pseudo-bulk calculations (y-axis). Colors represent density (yellow=high density; blue=low density). **(D)** Distribution of the fraction of labeled mRNA by averaging single cell GRAND-SLAM estimations (dark colored) or pseudo-bulk GRAND-SLAM calculations (light colored). Central dot is median; gray box bounds are 25th and 75th percentiles, upper and lower limits of whiskers are 1.5x interquartile ranges. Values outside of the upper and lower limits are defined as outliers, n=49 zygotic (blue) and 165 maternal (red). Characteristically low label incorporation rates and low per-cell number of reads by scRNA-Seq, limited the accuracy of labeled mRNA fraction estimated within single cells. Estimated labeled mRNA fraction for zygotic genes was often lower than expected, resulting in an unlabeled mRNA background. Aggregating single cells by stage and applying GRAND-SLAM estimation to these pseudo-bulk samples increased accuracy, bringing the estimated labeled mRNA fraction of zygotic genes to nearly 100%.

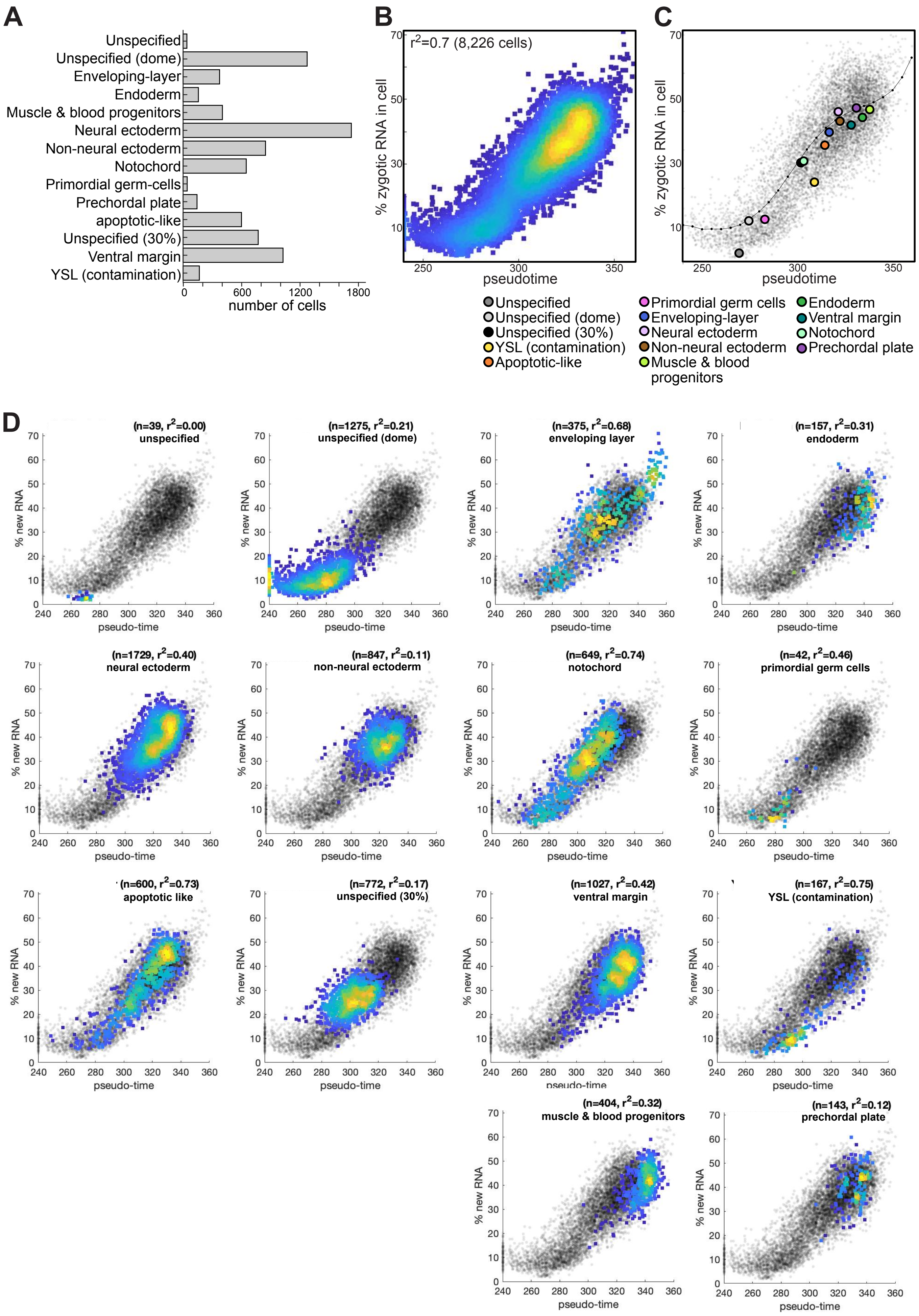

A
Unspecified
Unspecified (dome)
Enveloping-layer
Endoderm
Muscle & blood progenitors
Neural ectoderm
Non-neural ectoderm
Notochord
Primordial germ-cells
Prechordal plate
apoptotic-like
Unspecified (30%)
Ventral margin
YSL (contamination)
number of cells
B
$r^2$=0.7 (8,226 cells)
% zygotic RNA in cell
pseudotime
C
% zygotic RNA in cell
pseudotime
Unspecified
Unspecified (dome)
Unspecified (30%)
YSL (contamination)
Apoptotic-like
Primordial germ cells
Enveloping-layer
Neural ectoderm
Non-neural ectoderm
Muscle & blood progenitors
Endoderm
Ventral margin
Notochord
Prechordal plate
D
(n=39, $r^2$=0.00) unspecified
(n=1275, $r^2$=0.21) unspecified (dome)
(n=375, $r^2$=0.68) enveloping layer
(n=157, $r^2$=0.31) endoderm
(n=1729, $r^2$=0.40) neural ectoderm
(n=847, $r^2$=0.11) non-neural ectoderm
(n=649, $r^2$=0.74) notochord
(n=42, $r^2$=0.46) primordial germ cells
(n=600, $r^2$=0.73) apoptotic like
(n=772, $r^2$=0.17) unspecified (30%)
(n=1027, $r^2$=0.42) ventral margin
(n=167, $r^2$=0.75) YSL (contamination)
(n=404, $r^2$=0.32) muscle & blood progenitors
(n=143, $r^2$=0.12) prechordal plate
% new RNA
pseudo-time

Supplementary Figure 3: fraction of newly-transcribed zygotic mRNA in single cells is correlated to pseudotime

**(A)** Histogram of number of cells (x-axis) assigned to each of 12 distinct cell types that were defined in the data (y-axis). Endoderm A + B were merged. Cells labeled as YSL are likely a contamination that originated from the yolk syncytial layer. Unspecified cells are either from 30% epiboly or dome stages. **(B-D)** Scatter plots of pseudotime assigned to cells (x-axis, pseudo-min) and zygotic mRNA fraction in cells (y-axis). A cell specific fraction of zygotic RNA (across all genes) was calculated by the ratio of zygotic UMI counts to total UMI counts in the cell. **(B)** Colors represent density (yellow=high density; blue=low density) across all cells in dataset. Pearson R-squared value is indicated on plots. **(C)** Colored dots represent the averages of all cells in each cell type, and are colored by cell types (as indicated). Black dots and line represent a pseudotime sliding window average of fraction of zygotic mRNA in cells. **(D)** Black dots represent all cells in dataset. Colors in each plot represent density (yellow=high density; blue=low density) for cells from a specific cell type, as indicated. Pearson R-squared value and number of genes are indicated on each plot.

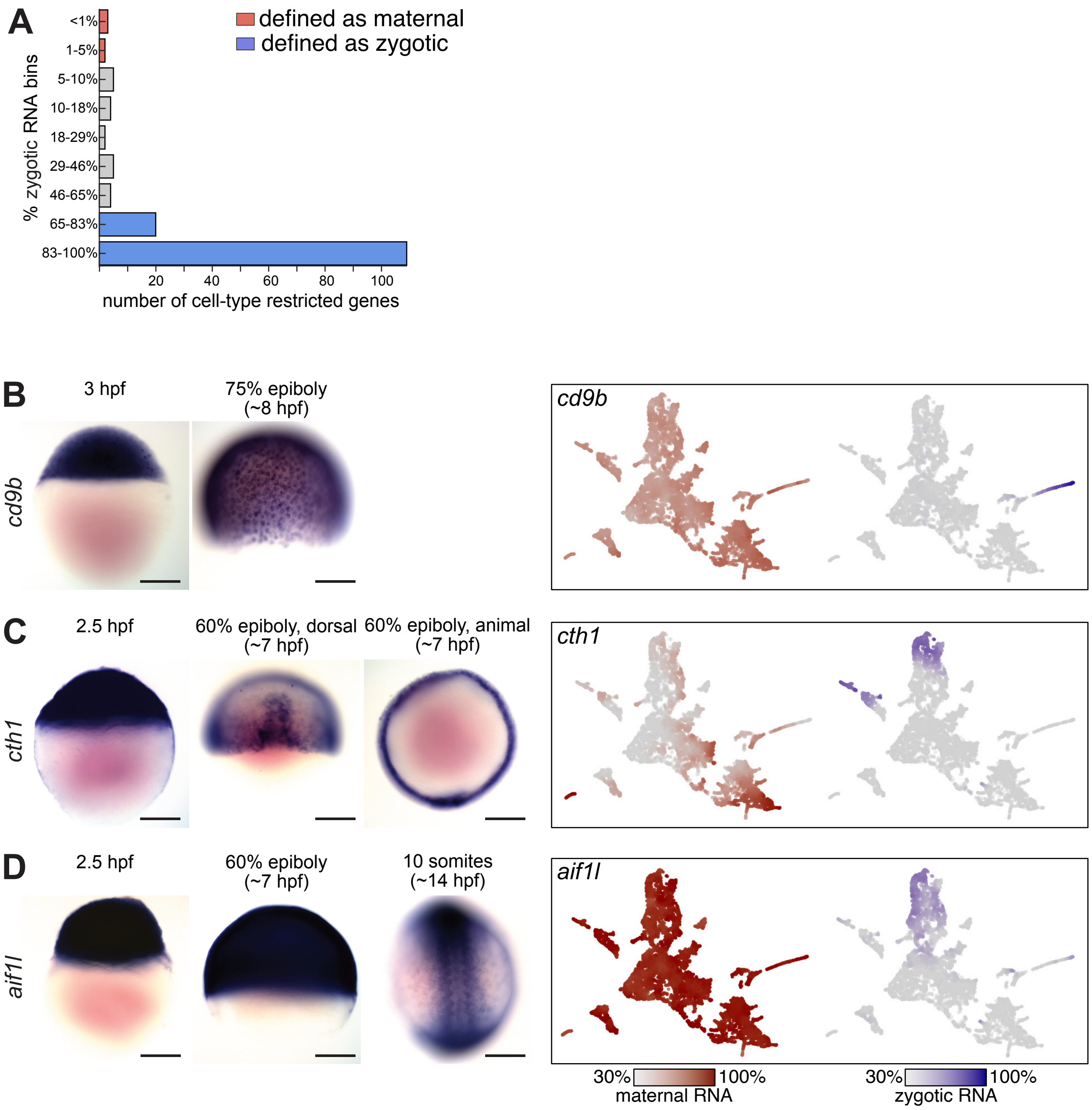

A
defined as maternal
defined as zygotic
% zygotic RNA bins
<1%
1-5%
5-10%
10-18%
18-29%
29-46%
46-65%
65-83%
83-100%
20
40
60
80
100
number of cell-type restricted genes
B
3 hpf
75% epiboly (~8 hpf)
cd9b
cd9b
C
2.5 hpf
60% epiboly, dorsal (~7 hpf)
60% epiboly, animal (~7 hpf)
cth1
cth1
D
2.5 hpf
60% epiboly (~7 hpf)
10 somites (~14 hpf)
aif1l
aif1l
30%
100%
maternal RNA
30%
100%
zygotic RNA

Supplementary Figure 4: variability in zygotic mRNA accumulation between genes

**(A)** Histogram of genes with cell type restricted expression (y-axis, number of genes) by their % of zygotic RNA quantile (9 quantiles, x-axis). Bars corresponding to top two bins are considered zygotic genes (blue, zygotic RNA fraction >65%) while bottom three bins are considered maternal genes (red, zygotic RNA fraction <5%). **(B-D)** Left: RNA *in situ* hybridization staining of zebrafish embryos for mRNAs of different embryonic genes, at specific developmental stages, as indicated. Right: Single cell expression of the same genes. All cells are plotted, and each cell is colored by the normalized expression of a gene's pre-existing (maternal) copies (red, left map) or newly-transcribed (zygotic) copies (blue, right map). Color-scale is gene specific, and scaled by its maximal total expression and its minimal 30% quantile of maternal and zygotic mRNA expression. Analyzed genes are indicated on plot. Genes are: **(B)** *cd9b*, **(C)** *cth1*, **(D)** *aif1l*.

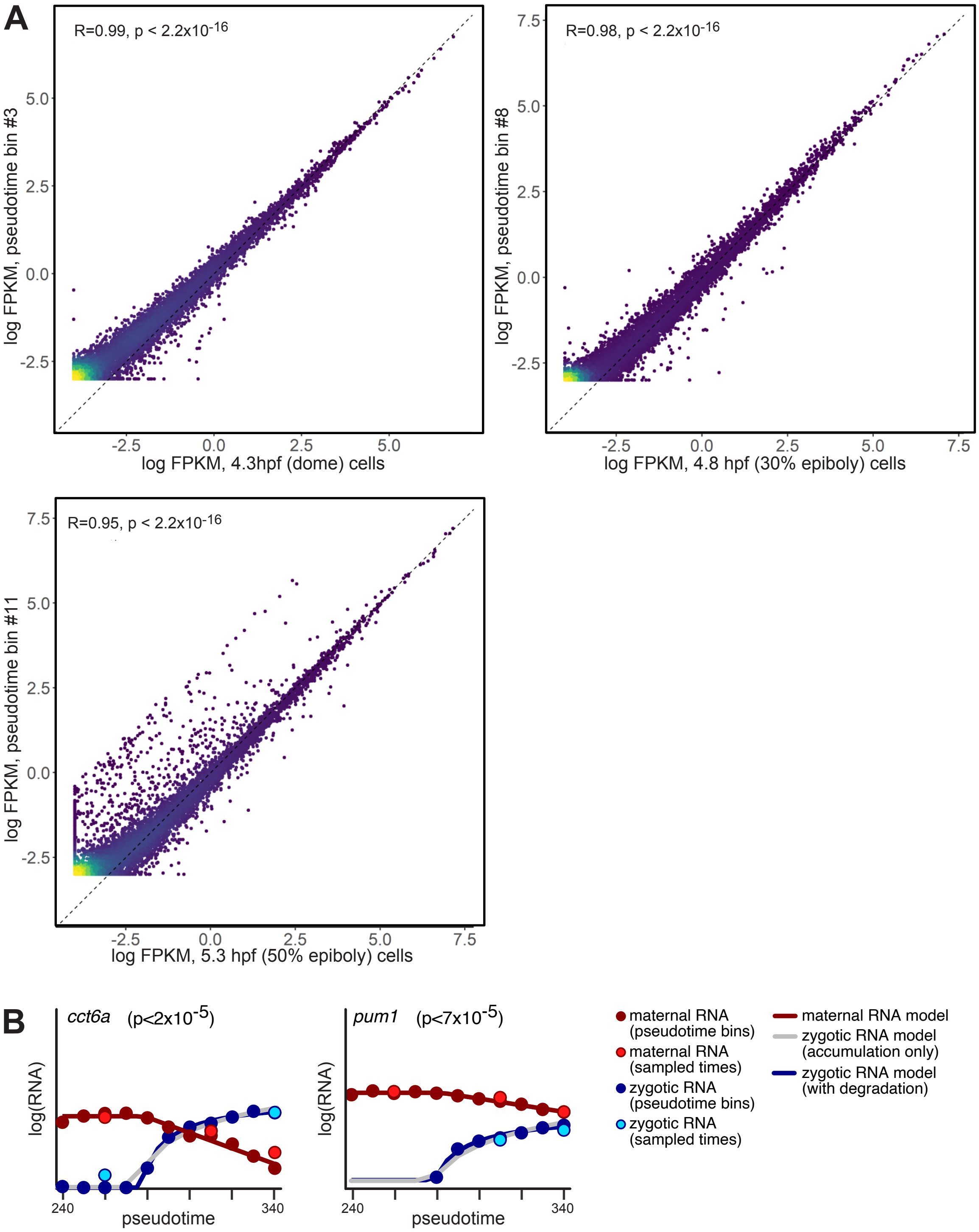

A
R=0.99, p < 2.2x10-16
log FPKM, pseudotime bin #3
log FPKM, 4.3hpf (dome) cells
R=0.98, p < 2.2x10-16
log FPKM, pseudotime bin #8
log FPKM, 4.8 hpf (30% epiboly) cells
R=0.95, p < 2.2x10-16
log FPKM, pseudotime bin #11
log FPKM, 5.3 hpf (50% epiboly) cells
-2.5
0.0
2.5
5.0
7.5
B
cct6a (p<2x10-5)
pum1 (p<7x10-5)
log(RNA)
pseudotime
240
340
maternal RNA (pseudotime bins)
maternal RNA (sampled times)
zygotic RNA (pseudotime bins)
zygotic RNA (sampled times)
maternal RNA model
zygotic RNA model (accumulation only)
zygotic RNA model (with degradation)

Supplementary Figure 5: modeling the dynamics of the maternal and zygotic transcriptomes

**(A)** Scatter plots of RNA expression ($log_2$) 3 embryonic developmental stages (x-axis; left: dome, 4.3 hpf; middle: 30% epiboly, 4.8 hpf; right: 50% epiboly, 5.3 hpf) and in 3 pseudotime bins (y-axis; left: bin #3; middle: bin #8; right: bin #11). Colors represent density (yellow=high density; blue=low density). Pearson R values are indicated on plots. **(B)** Model fits (solid lines) to interpolated zygotic (blue dots) and maternal (red dots) expression levels (y-axis, $log_2$ scale) across 11 pseudotime (x-axis) bins for genes which significantly rejected the zygotic accumulation model (gray line) in favor of the more complex model with zygotic degradation (blue line). Gene name and likelihood ratio test p-values (for selecting between simpler or complex model) are indicated on top. Light blue and light red dots represent estimated zygotic and maternal mRNA levels, respectively, for three sampled timepoints (4.3 hpf, 4.8 hpf and 5.3 hpf).

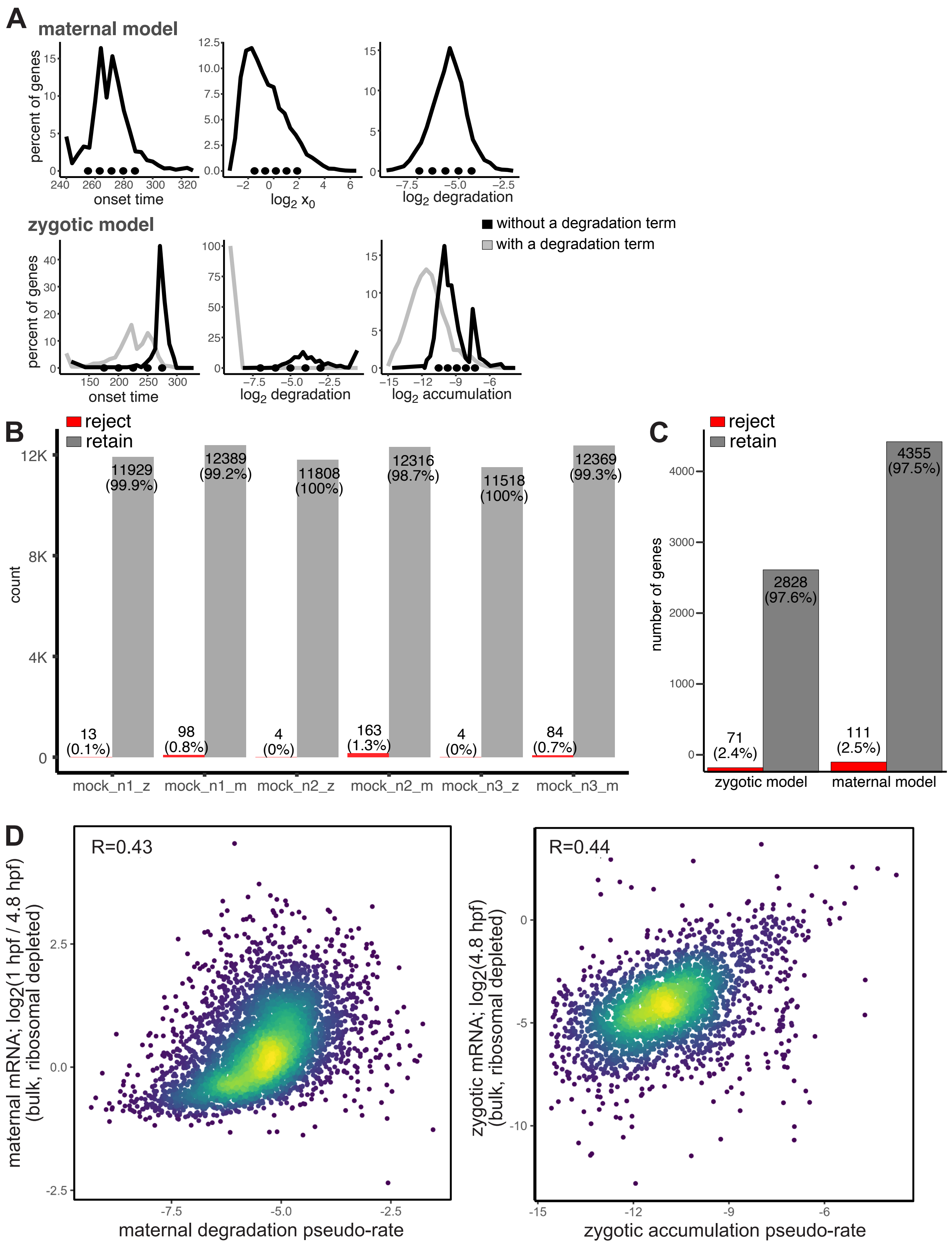

A
maternal model
percent of genes
onset time
log2 x0
log2 degradation
zygotic model
percent of genes
onset time
log2 degradation
log2 accumulation
without a degradation term
with a degradation term
B
reject
retain
count
11929 (99.9%)
12389 (99.2%)
11808 (100%)
12316 (98.7%)
11518 (100%)
12369 (99.3%)
13 (0.1%)
98 (0.8%)
4 (0%)
163 (1.3%)
4 (0%)
84 (0.7%)
mock_n1_z
mock_n1_m
mock_n2_z
mock_n2_m
mock_n3_z
mock_n3_m
C
reject
retain
number of genes
2828 (97.6%)
4355 (97.5%)
71 (2.4%)
111 (2.5%)
zygotic model
maternal model
D
R=0.43
maternal mRNA; log2(1 hpf / 4.8 hpf) (bulk, ribosomal depleted)
maternal degradation pseudo-rate
R=0.44
zygotic mRNA; log2(4.8 hpf) (bulk, ribosomal depleted)
zygotic accumulation pseudo-rate

Supplementary Figure 6: validations of kinetic models for maternal and zygotic mRNA expression dynamics

**(A)** Selection of parameter values for simulation studies for each of 6 model parameters. Shown are distributions of parameter values that were fitted by the maternal or zygotic RNA expression models to genes. Top: maternal decay model parameters (y-axis, density; x-axis left to right: degradation onset time (pseudo-min), initial expression level ($log_2$), degradation pseudo-rate ($log_2$ 1/pseudo-min)). Bottom: zygotic accumulation model parameters, either without a degradation term (black), or with a degradation term (gray) (y-axis, density; x-axis left to right: transcription onset time (pseudo-min), degradation pseudo-rate ($log_2$), accumulation pseudo-rate ($log_2$ transcripts/pseudo-min)). **(B)** Histogram of number of mock generated samples (y-axis) that retained (gray) or rejected (red) the fitted model by a goodness-of-fit test in each of 3 simulation experiments (x-axis) with increasing noise levels: 50% of standard deviation (as estimated in replicated samples) (n1), 100% of standard deviation (n2) and 150% of standard deviation (n3). Number of samples is indicated. **(C)** Histogram of number of genes (y-axis) that retained (gray) or rejected (red) the fitted model (x-axis) by a goodness-of-fit test (chi-square p-value). Number of genes is indicated. **(D)** Scatter plots comparing kinetic parameters that were estimated for each gene by the model (x-axis; left: maternal mRNA degradation pseudo-rate, right: zygotic mRNA accumulation pseudo-rate) to fold-change of mRNA that was estimated in ribosomal depleted bulk samples (y-axis, $log_2$; left: maternal mRNA, right: zygotic mRNA). Fold change of maternal mRNA was estimated by ratio of expression in 1 hpf (4-cell) to 4.8 hpf (30% epiboly) samples. Fold change of zygotic mRNA was estimated by the expression in 4.8 hpf (30% epiboly) sample, since zygotic mRNA expression at the 1 hpf sample was below detection. Colors represent density (yellow=high density; blue=low density). Pearson R values are indicated on plots.

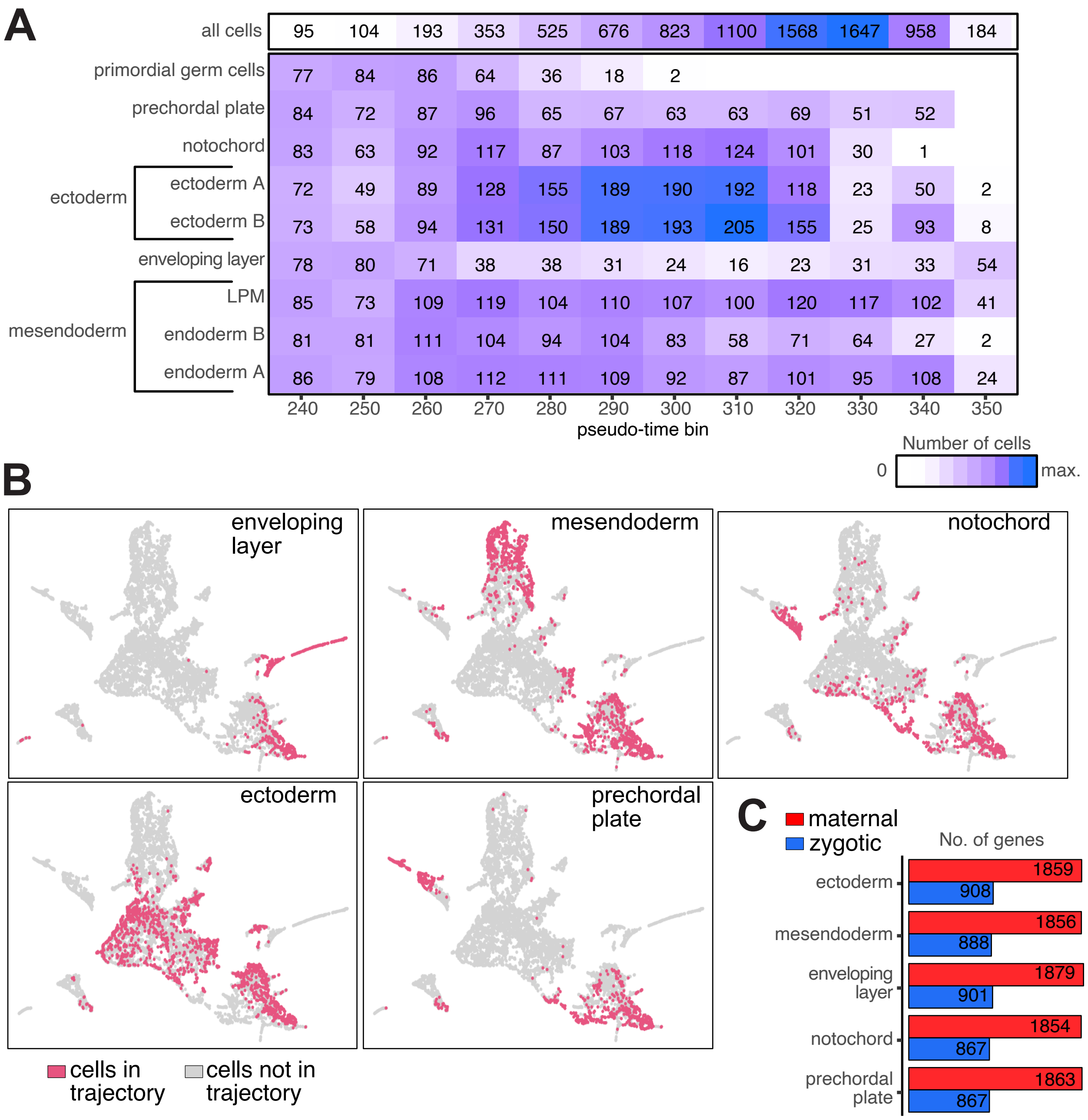
A
all cells 95 104 193 353 525 676 823 1100 1568 1647 958 184
primordial germ cells 77 84 86 64 36 18 2
prechordal plate 84 72 87 96 65 67 63 63 69 51 52
notochord 83 63 92 117 87 103 118 124 101 30 1
ectoderm
ectoderm A 72 49 89 128 155 189 190 192 118 23 50 2
ectoderm B 73 58 94 131 150 189 193 205 155 25 93 8
enveloping layer 78 80 71 38 38 31 24 16 23 31 33 54
mesendoderm
LPM 85 73 109 119 104 110 107 100 120 117 102 41
endoderm B 81 81 111 104 94 104 83 58 71 64 27 2
endoderm A 86 79 108 112 111 109 92 87 101 95 108 24
240 250 260 270 280 290 300 310 320 330 340 350
pseudo-time bin
Number of cells
0
max.
B
enveloping layer
mesendoderm
notochord
ectoderm
prechordal plate
cells in trajectory
cells not in trajectory
C
maternal
zygotic
No. of genes
ectoderm 1859 908
mesendoderm 1856 888
enveloping layer 1879 901
notochord 1854 867
prechordal plate 1863 867

Supplementary Figure 7: modeling mRNA regulation within developmental trajectories

**(A)** Number of cells in each subset of cells (rows) assigned to 11 pseudotime bins (columns). Color-scale represents number of cells (white = no cells; dark purple = maximal number of cells). Number of cells is indicated. **(B)** Single-cell UMAP projection with cells assigned to each of 5 developmental trajectories (as indicated on map) colored in red. **(C)** Histogram of number of genes (y-axis) analyzed in each developmental trajectory (x-axis). Red: maternal genes, Blue: zygotic genes.

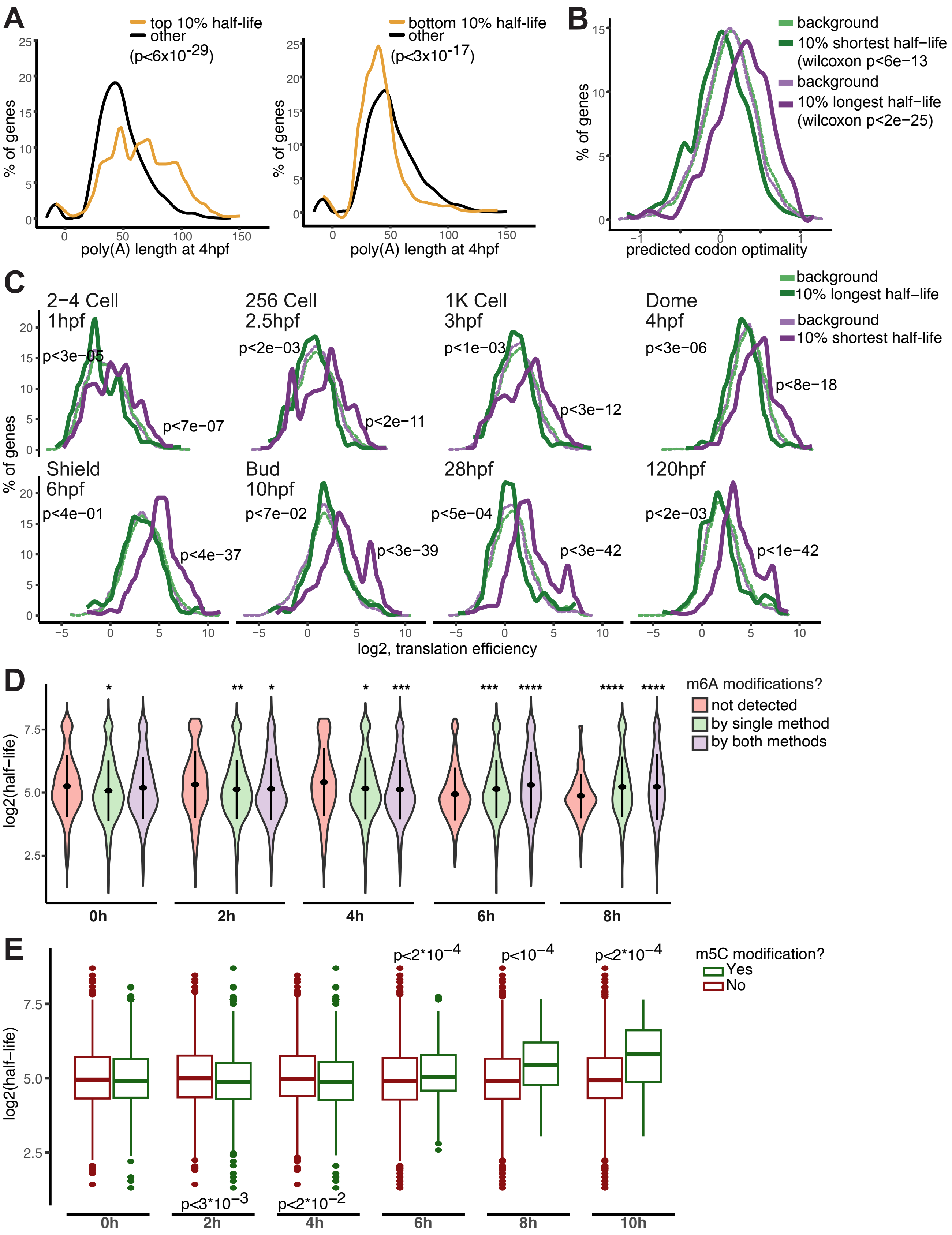

A
top 10% half-life
other
(p<6x10-29)
bottom 10% half-life
other
(p<3x10-17)
% of genes
poly(A) length at 4hpf
B
background
10% shortest half−life
(wilcoxon p<6e−13)
background
10% longest half−life
(wilcoxon p<2e−25)
% of genes
predicted codon optimality
C
background
10% longest half−life
background
10% shortest half−life
2−4 Cell 1hpf
p<3e−05
p<7e−07
256 Cell 2.5hpf
p<2e−03
p<2e−11
1K Cell 3hpf
p<1e−03
p<3e−12
Dome 4hpf
p<3e−06
p<8e−18
Shield 6hpf
p<4e−01
p<4e−37
Bud 10hpf
p<7e−02
p<3e−39
28hpf
p<5e−04
p<3e−42
120hpf
p<2e−03
p<1e−42
% of genes
log2, translation efficiency
D
m6A modifications?
not detected
by single method
by both methods
log2(half−life)
0h
2h
4h
6h
8h
E
m5C modification?
Yes
No
log2(half−life)
p<3*10−3
p<2*10−2
p<2*10−4
p<10−4
p<2*10−4
0h
2h
4h
6h
8h
10h

Supplementary Figure 8: sequence enrichments associated with regulatory rates of mRNA degradation

**(A)** Frequency (y-axis) of poly(A) tail length (x-axis) as measured by [2]. Left: for genes with pseudo-half-life at the top 10% (yellow) or other (black). Right: for genes with pseudo-half-life at the bottom 10% (yellow) or other (black). P-value of a one-sided Kolmogorov-Smirnov test (FDR <1%) is indicated. **(B)** Frequency (y-axis) of codon optimality predictions by [3]. For 10% of genes with the longest pseudo-half-life (purple, solid) or other (purple, dashed), and for 10% of genes with shortest pseudo-half-life (green, solid) or other (green, dashed). P-value of a wilcoxon rank sum test is indicated. **(C)** Frequency (y-axis) of mean transcript translation efficiency (x-axis, $\log_2$) as measured by [4] at different zebrafish developmental stages. Colors as in (B). P-value of a one-sided Kolmogorov-Smirnov test (FDR <1%) is indicated. **(D)** Distribution of predicted mRNA pseudo-half-lives for genes with evidence for $m^6A$ modification by [5] in both m6A-seq and m6A-CLIP-seq (purple), in only one of the two datasets (green), or in neither (orange). Central dot represents the mean, lines extend to mean +/1 standard-deviation, n=1128, 1455, 849, 1026, 932 by single method per time; 1620, 1361, 2112, 1446, 1742 by both methods per time; 551, 483, 338, 827, 625 for not detected per time. P-values represent a Wilcoxon rank sum test of the neither group with each of the other 3 groups. Significance is indicated by *: p <= 0.05, **: p <= 0.01, ***: p <= 0.001, ****: p <= 0.0001. **(E)** Distribution of pseudo-half-life (y-axis, $\log_2$) in each timepoint analyzed (x-axis) with evidence for an $m^5C$ modification (green) or without (red). A gene was considered $m^5C$ modified if it contained a location with an $m^5C$ level >= 0.25, as measured by [6]. Central line represents the median, box edges are 25th and 75th percentiles, whiskers extend to largest/smallest value except outliers; outlier points are plotted individually, n=1217, 1092, 1138, 1444, 1681 for no modification per time; 595, 729, 674, 368, 131 for with modification per time. P-values represent a one-sided Kolomogorov-Smirnoff test between half-life of modified and non-modified genes at each timepoint.

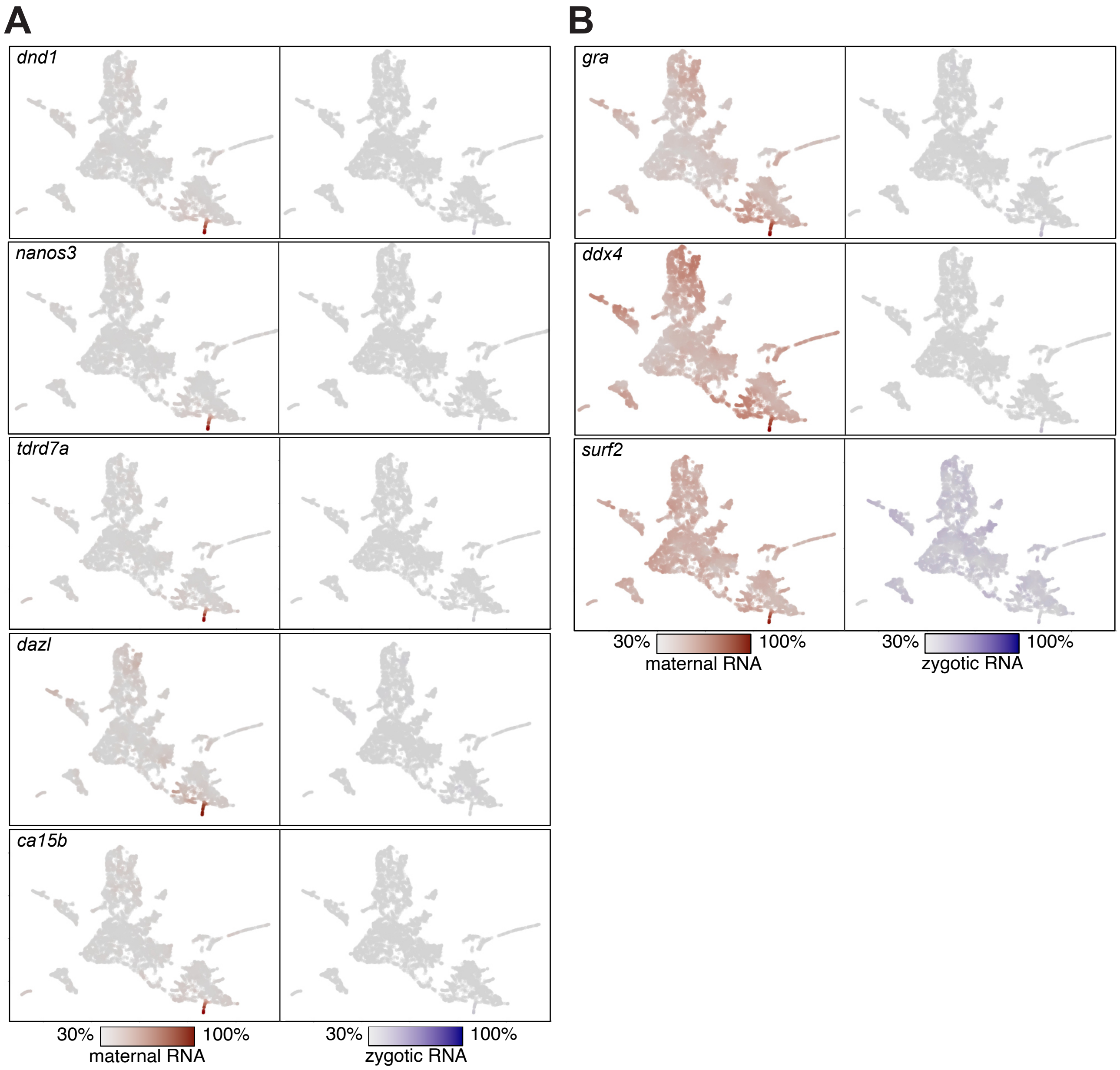

A
B
dnd1
nanos3
tdrd7a
dazl
ca15b
gra
ddx4
surf2
30%
100%
maternal RNA
30%
100%
zygotic RNA

Supplementary Figure 9: two patterns of germ-cell specific maternally expressed genes

Single cell expression of 8 germ-cell specific expressed genes across all 8,226 collected single cells. All cells are plotted, and each cell is colored by the normalized expression of a gene's maternal copies (red, left) or zygotic copies (blue, right). Color-scale is gene specific, and scaled by its maximal total expression, and its minimal 30% quantile of maternal and zygotic RNA expression. Analyzed genes are indicated on plot. Plotted genes show cell lineage specificity within germ-cells with either **(A)** low somatic background or **(B)** high somatic background.

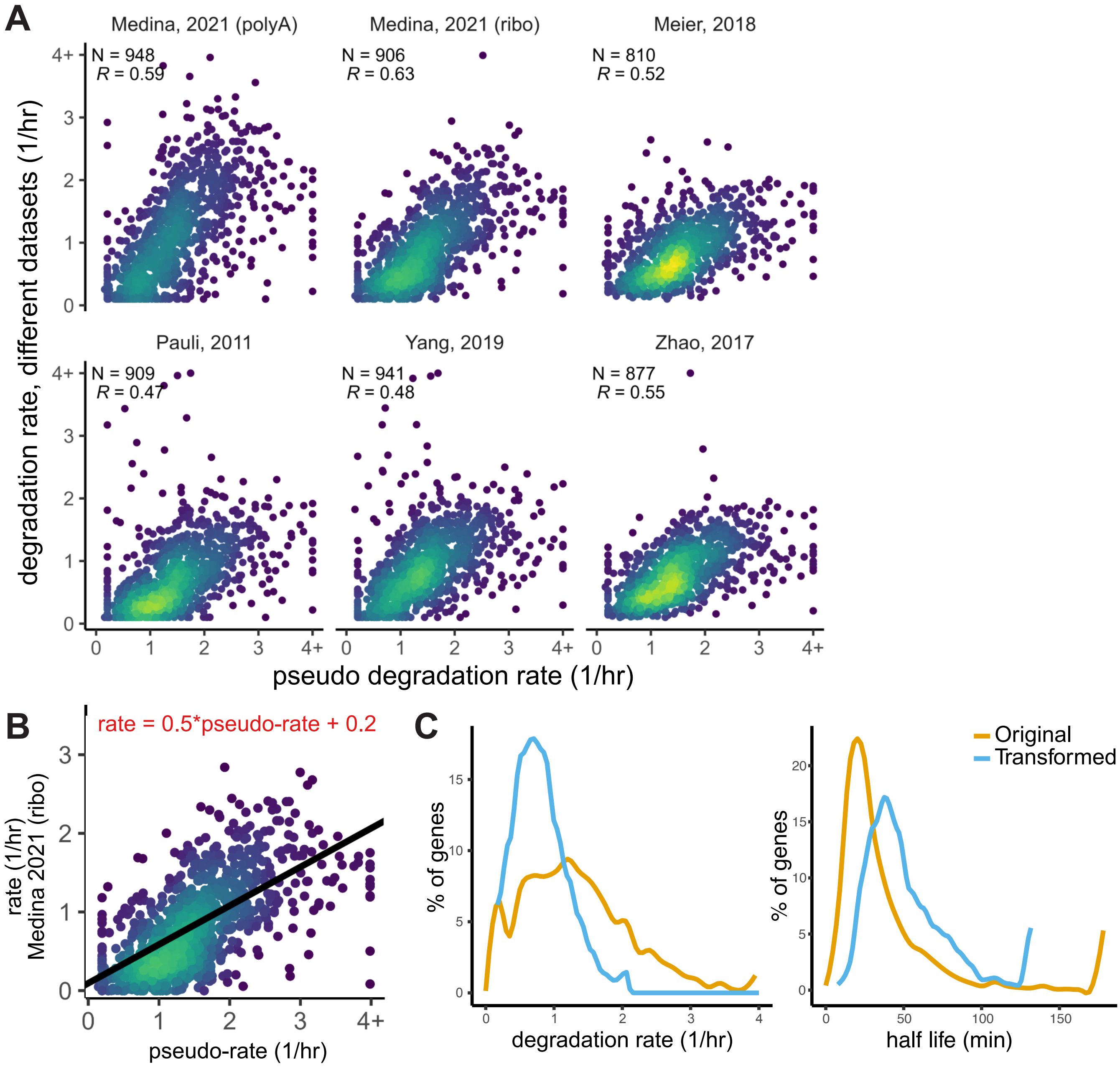

A
Medina, 2021 (polyA)
N = 948
R = 0.59
Medina, 2021 (ribo)
N = 906
R = 0.63
Meier, 2018
N = 810
R = 0.52
Pauli, 2011
N = 909
R = 0.47
Yang, 2019
N = 941
R = 0.48
Zhao, 2017
N = 877
R = 0.55
degradation rate, different datasets (1/hr)
pseudo degradation rate (1/hr)
B
rate = 0.5*pseudo-rate + 0.2
rate (1/hr)
Medina 2021 (ribo)
pseudo-rate (1/hr)
C
% of genes
degradation rate (1/hr)
Original
Transformed
half life (min)

Supplementary Figure 10: correlation of pseudo and absolute rates of maternal genes' degradation

**(A)** Correlation between degradation rates calculated for maternal-only genes in several RNA-Seq datasets (y-axis; name of dataset indicated on top of each plot) and pseudo degradation rates from current analysis (x-axis) (colorscale indicates density; yellow: denser, purple: sparser). Number of genes (N) and Pearson correlation (R) indicated in top left of each plot. **(B)** Correlation between degradation rates calculated for maternal-only genes from temporal total-RNA-Seq data [7], and pseudo degradation rates from current analysis (x-axis) (colorscale indicates density; yellow: denser, purple: sparser). Degradation rates were calculated for maternal-only genes as previously described [8]. Black line represents a linear regression model to connect the two estimates. Model parameters (red) indicated on top. **(C)** Frequency (y-axis; % of genes) of degradation rate (left) and half-life (right) of the original pseudo-rates (orange) and rates after correction using the linear model from (B) (blue). This analysis suggests that pseudo-rates over estimate absolute rates by 2-fold, and are shifted by an additive factor of 0.2 (1/hr). Corrected pseudo-rates could reflect more reliable absolute estimates of mRNA degradation rates in our data.

| | **Start_lower** | **Start_upper** | **lower** | **Upper** |
|---|---|---|---|---|
| **Maternal $\beta$** | Half-life = 2 hrs | Half-life = 15 min. | Half-life = 10 hrs | Half-life = 1 min. |
| **Maternal $x_0$** | Min. expression | Max. expression | -4 | 30 |
| **Maternal $d$** | First time point with expression | Last time point with expression | First time point with expression | Inf |
| **Zygotic $\alpha$** | -15 | 1 | -20 | $10 - \log_2 60$ |
| **Zygotic $\beta$** | Half-life = 2 hrs | Half-life = 15 min. | Half-life = 10 hrs | Half-life = 1 min. |
| **Zygotic $d$** | 140 min. | First time point with expression + 60 min. | 120 min. | Inf |

**Supplementary Table 1**: Parameters bounds for non-linear model fits

Parameters bounds for non-linear models used in fitting of kinetic models of maternal and zygotic mRNA expression dynamics.

## References


1. Farrell, J. A. *et al.* Single-cell reconstruction of developmental trajectories during zebrafish embryogenesis. *Science* **360**, eaar3131 (2018).
2. Subtelny, A. O., Eichhorn, S. W., Chen, G. R., Sive, H. & Bartel, D. P. Poly(A)-tail profiling reveals an embryonic switch in translational control. *Nature* **508**, 66–71 (2014).
3. Diez, M. *et al.* iCodon customizes gene expression based on the codon composition. *Sci Rep* **12**, 12126 (2022).
4. Chew, G.-L. *et al.* Ribosome profiling reveals resemblance between long non-coding RNAs and 5' leaders of coding RNAs. *Development* **140**, 2828–2834 (2013).
5. Zhao, B. S. *et al.* m6A-dependent maternal mRNA clearance facilitates zebrafish maternal-to-zygotic transition. *Nature* **542**, 475–478 (2017).
6. Liu, J. *et al.* Developmental mRNA m5C landscape and regulatory innovations of massive m5C modification of maternal mRNAs in animals. *Nat Commun* **13**, 2484 (2022).
7. Medina-Muñoz, S. G. *et al.* Crosstalk between codon optimality and cis-regulatory elements dictates mRNA stability. *Genome Biol* **22**, 14 (2021).
8. Rabani, M., Pieper, L., Chew, G.-L. & Schier, A. F. A Massively Parallel Reporter Assay of 3' UTR Sequences Identifies In Vivo Rules for mRNA Degradation. *Mol Cell* **68**, 1083-1094.e5 (2017).
