# Supplementary material for: Cell-type-specific mRNA transcription and degradation kinetics in zebrafish embryogenesis from metabolically labeled single-cell RNA-seq
Source: Nat Commun. 2024 Apr 10;15:3104. doi: 10.1038/s41467-024-47290-9 (PMC11006943; doi:10.1038/s41467-024-47290-9)
Supplement: Supplementary file 3 — Description of additional supplementary files [file 41467_2024_47290_MOESM3_ESM.pdf]

DESCRIPTION OF ADDITIONAL SUPPLEMENTARY FILES DOCUMENT

Supplementary Data 1: List of 436 cell-type-enriched genes.

A one-sided Kolmogorov-Smirnoff test was used to define these genes and 1% Bonferroni multiple testing correction.

Supplementary Data 2: List of functional enrichments in groups A-D from Figure 5.

A 5% g:SCS multiple testing correction method was used.

Supplementary Data 3: List of 149 genes with trajectory-specific mRNA dynamics.

A likelihood ratio test was used to define these genes and 1% FDR multiple testing correction.
