# Supplementary material for: Cell-type-specific mRNA transcription and degradation kinetics in zebrafish embryogenesis from metabolically labeled single-cell RNA-seq
Source: Nat Commun. 2024 Apr 10;15:3104. doi: 10.1038/s41467-024-47290-9 (PMC11006943; doi:10.1038/s41467-024-47290-9)
Supplement: Supplementary file 7 — Reporting Summary [file 41467_2024_47290_MOESM7_ESM.pdf]

nature portfolio

Corresponding author(s): Jeffrey A. Farrell, Michal Rabani

Last updated by author(s): March 3, 2024

# Reporting Summary

Nature Portfolio wishes to improve the reproducibility of the work that we publish. This form provides structure for consistency and transparency in reporting. For further information on Nature Portfolio policies, see our Editorial Policies and the Editorial Policy Checklist.

## Statistics

For all statistical analyses, confirm that the following items are present in the figure legend, table legend, main text, or Methods section.

| n/a | Confirmed | |
|---|---|---|
| ☐ | ☒ | The exact sample size (*n*) for each experimental group/condition, given as a discrete number and unit of measurement |
| ☐ | ☒ | A statement on whether measurements were taken from distinct samples or whether the same sample was measured repeatedly |
| ☐ | ☒ | The statistical test(s) used AND whether they are one- or two-sided<br>*Only common tests should be described solely by name; describe more complex techniques in the Methods section.* |
| ☒ | ☐ | A description of all covariates tested |
| ☐ | ☒ | A description of any assumptions or corrections, such as tests of normality and adjustment for multiple comparisons |
| ☐ | ☒ | A full description of the statistical parameters including central tendency (e.g. means) or other basic estimates (e.g. regression coefficient) AND variation (e.g. standard deviation) or associated estimates of uncertainty (e.g. confidence intervals) |
| ☐ | ☒ | For null hypothesis testing, the test statistic (e.g. *F*, *t*, *r*) with confidence intervals, effect sizes, degrees of freedom and *P* value noted<br>*Give P values as exact values whenever suitable.* |
| ☒ | ☐ | For Bayesian analysis, information on the choice of priors and Markov chain Monte Carlo settings |
| ☒ | ☐ | For hierarchical and complex designs, identification of the appropriate level for tests and full reporting of outcomes |
| ☐ | ☒ | Estimates of effect sizes (e.g. Cohen's *d*, Pearson's *r*), indicating how they were calculated |

*Our web collection on statistics for biologists contains articles on many of the points above.*

## Software and code

Policy information about availability of computer code

| | |
|---|---|
| Data collection | Sequencing libraries were analyzed using Nextseq 500/550 and Novaseq 6000 analysis software (Illumina). Images were collected using the Nikon Elements software that is standard for Nikon confocals or ZEN that is standard for Zeiss microscopes. |
| Data analysis | Drop-Seq data was analyzed using Drop-seq tools v1.12 and URD software. Bulk RNA was analyzed using cutadapt/3.5, STAR/2.7.10a, GRAND-SLAM/2.0.5f. Other metabolic labeling signals were analyzed using GRAND3_3.0.0 software. Other custom analysis and modeling were performed using Matlab (R2018b) and R scripts (mostly R4/4.1.3). R packages used include data.table, ggplot2, dplyr, tidyr, reshape2, tidyverse, stringr, seqinr, kmer, adegenet, Seurat, shiny, minpack.lm, nls.multstart, multidplyr and BiocManager. Additionally, other packages were used sporadically for specific analyses as needed. Image analysis was performed using Fiji (ImageJ), and the series of commands used for analysis is described in the Methods. |

For manuscripts utilizing custom algorithms or software that are central to the research but not yet described in published literature, software must be made available to editors and reviewers. We strongly encourage code deposition in a community repository (e.g. GitHub). See the Nature Portfolio guidelines for submitting code & software for further information.

## Data

Policy information about availability of data

All manuscripts must include a data availability statement. This statement should provide the following information, where applicable:

- Accession codes, unique identifiers, or web links for publicly available datasets
- A description of any restrictions on data availability
- For clinical datasets or third party data, please ensure that the statement adheres to our policy

Sequencing data generated in this study have been deposited in the NCBI Gene Expression Omnibus, under accessions GSE224113 (bulk RNA-Seq) and GSE224918 (single-cell RNA-Seq) and is freely available. Raw microscopy data for figures presented in this study is available from Zenodo (doi:10.5281/zenodo.10080888). In addition, our portal (https://liorf.shinyapps.io/zebrafish_single_cell_regulation) provides the scientific community with ready access to our data and analysis results. Published datasets used in this study are available in the NCBI Gene Expression Omnibus, under accessions GSE106587 (for expression comparison after 4sUTP injections), GSE52809 (for poly(A) tail lengths), GSE46512 (for ribosome profiling), GSE79213 (for m6A analysis), GSE127780 (for m5C analysis), GSE79213, GSE84601, GSE148391, GSE120643, GSE32898 and GSE56977 (for maternal genes' degradation rates comparisons).

## Research involving human participants, their data, or biological material

Policy information about studies with human participants or human data. See also policy information about sex, gender (identity/presentation), and sexual orientation and race, ethnicity and racism.

| | |
|---|---|
| Reporting on sex and gender | N/A |
| Reporting on race, ethnicity, or other socially relevant groupings | N/A |
| Population characteristics | N/A |
| Recruitment | N/A |
| Ethics oversight | N/A |

Note that full information on the approval of the study protocol must also be provided in the manuscript.

# Field-specific reporting

Please select the one below that is the best fit for your research. If you are not sure, read the appropriate sections before making your selection.

☒ Life sciences ☐ Behavioural & social sciences ☐ Ecological, evolutionary & environmental sciences

For a reference copy of the document with all sections, see nature.com/documents/nr-reporting-summary-flat.pdf

# Life sciences study design

All studies must disclose on these points even when the disclosure is negative.

| | |
|---|---|
| Sample size | For single-cell RNA Sequencing we did not perform sample size calculation. We include 2 biological replicates for 2 out of 3 developmental stages. For the first replicate, a total of 100 injected embryos were randomly collected per sample. For the second replicate a total of 70-75 embryos were randomly collected per sample. For bulk RNA-Seq, 25 embryos were collected per sample. For images, sample size is indicated for each experiment in the quantification; power analysis was not performed. |
| Data exclusions | For single-cell data, only cells with expression of at least 500 genes were retained. For images, embryos were excluded if they were mounted in an inappropriate orientation to visualize the mRNAs of interest. |
| Replication | For single-cell RNA Sequencing we include 2 biological replicates for 2 out of 3 developmental stages. We indicate replications for each experiment, and ensure reproducibility. |
| Randomization | Embryos were randomly collected from natural mating of zebrafish. |
| Blinding | Blinding was not used in this study as single-cell and bulk RNA-Seq were not affected by investigators' expectations. |

# Reporting for specific materials, systems and methods

We require information from authors about some types of materials, experimental systems and methods used in many studies. Here, indicate whether each material, system or method listed is relevant to your study. If you are not sure if a list item applies to your research, read the appropriate section before selecting a response.

## Materials & experimental systems

| n/a | Involved in the study |
| --- | --- |
| ☒ | ☐ Antibodies |
| ☒ | ☐ Eukaryotic cell lines |
| ☒ | ☐ Palaeontology and archaeology |
| ☐ | ☒ Animals and other organisms |
| ☒ | ☐ Clinical data |
| ☒ | ☐ Dual use research of concern |
| ☒ | ☐ Plants |

## Methods

| n/a | Involved in the study |
| --- | --- |
| ☒ | ☐ ChIP-seq |
| ☒ | ☐ Flow cytometry |
| ☒ | ☐ MRI-based neuroimaging |

# Animals and other research organisms

Policy information about studies involving animals; ARRIVE guidelines recommended for reporting animal research, and Sex and Gender in Research

| | |
| --- | --- |
| Laboratory animals | wild-type AB/TL zebrafish (Danio Rerio) aged 6-18 months |
| Wild animals | N/A |
| Reporting on sex | sex was not considered in study design, as zebrafish sex cannot be determined at early developmental stages. |
| Field-collected samples | N/A |
| Ethics oversight | All protocols and procedures involving zebrafish were approved by the Harvard University/Faculty of Arts and Sciences Standing Committee on the Use of Animals in Research and Teaching (IACUC; Protocol #25-08), the Hebrew University Ethics Committee (IACUC; Protocol #NS-15859), and the National Institute of Child Health and Human Development (ACUC; Protocols 20-001, 23-001). |

Note that full information on the approval of the study protocol must also be provided in the manuscript.

# Plants

| | |
| --- | --- |
| Seed stocks | N/A |
| Novel plant genotypes | N/A |
| Authentication | N/A |
